# An individual patient data meta-analysis of adjuvant therapy with uracil–tegafur (UFT) in patients with curatively resected rectal cancer

**DOI:** 10.1038/sj.bjc.6603686

**Published:** 2007-03-20

**Authors:** J Sakamoto, C Hamada, S Yoshida, S Kodaira, M Yasutomi, T Kato, K Oba, H Nakazato, S Saji, Y Ohashi

**Affiliations:** 1 Meta-Analysis Group of the Japanese Society for Cancer of the Colon and Rectum; Secretariat, Department of Epidemiological & Clinical Research Information Management, Kyoto University, Graduate School of Medicine, Kyoto, Japan

**Keywords:** rectal cancer, UFT, adjuvant chemotherapy, randomised clinical trials, individual patient data meta-analysis

## Abstract

Uracil–Tegafur (UFT), an oral fluorinated pyrimidine chemotherapeutic agent, has been used for adjuvant chemotherapy in curatively resected colorectal cancer patients. Past trials and meta-analyses indicate that it is somewhat effective in extending survival of patients with rectal cancer. The objective of this study was to perform a reappraisal of randomised clinical trials conducted in this field. We designed an individual patient-based meta-analysis of relevant clinical trials to examine the benefit of UFT for curatively resected rectal cancer in terms of overall survival (OS), disease-free survival (DFS), and local relapse-free survival (LRFS). We analysed individual patient data of five adjuvant therapy randomised clinical trials for rectal cancer, which met the predetermined inclusion criteria. These five trials had a combined total of 2091 patients, UFT as adjuvant chemotherapy compared to surgery-alone, 5-year follow-up, intention-to-treat-based analytic strategy, and similar endpoints (OS and DFS). In a pooled analysis, UFT had significant advantage over surgery-alone in terms of both OS (hazard ratio, 0.82; 95% confidence interval (CI), 0.70–0.97; *P*=0.02) and DFS (hazard ratio, 0.73; 95%CI, 0.63–0.84; *P*<0.0001). This individual patient-based meta-analysis demonstrated that oral UFT significantly improves both OS and DFS in patients with curatively resected rectal cancer.

Colorectal cancer accounts for 10–15% of all cancers and is the second leading cause of cancer deaths in developed countries (Pisani *et al*, 1993). In Japan alone, nearly 56 000 new cases are diagnosed and this disease causes 36 000 deaths every year (Statistics and information department, Ministry of Health and Welfare, 1996). Surgical treatment is the primary management of colorectal cancers, with 75–80% of the patients being operable at the time of diagnosis (Boring *et al*, 1991; Vernaba *et al*, 1994). However, even if a curative resection is performed, those patients with regional lymph node involvement (Dukes' C, Stage III) have a 40–50% 5-year survival rate.

Recently, in the field of Stage III colon cancer treatment, adjuvant chemotherapy by 5-fluorouracil (5-FU)/levamisole was proved to be superior to surgery-alone therapy, and then various 5-FU/leucovorin (LV) regimens were confirmed to be effective from the results of numerous large-scale randomised trials and from the pooled analysis of clinical trials (Wolmark *et al*, 1993; International Multicentre Pooled Analysis of Colon Cancer Trials (IMPACT) investigators, 1995; O'Connell *et al*, 1997). In 2004, results from the Multicenter International Study of Oxaliplatin/5-FU/Leucovorin in the Adjuvant Treatment of Colon Cancer (MOSAIC) trial demonstrated that combination chemotherapy with 5-FU/LV (de Gramont regimen) plus oxaliplatin was significantly superior to 5-FU/LV alone (André *et al*, 2004). With regard to adjuvant chemotherapy for colon cancer, therefore, solid evidence has been accumulated from relevant clinical trials, and steady evolution of the new treatment modalities has been achieved.

However, the situation is still uncertain focusing on adjuvant therapy for rectal cancer. Despite apparently curative surgery, rectal cancer recurs in more than 55% of the patients, including local recurrence rates of 25% (Vernaba *et al*, 1994). Despite the recommendation of the consensus conference by the National Institute of Health (NIH consensus conference, 1990) that concluded that adjuvant radiotherapy and chemotherapy should be given to all patients with locally advanced rectal cancer, recent findings by a large-scale randomised trial and meta-analysis have failed to prove significant benefit of radiotherapy for survival (Fisher *et al*, 1988; Vernaba *et al*, 1994). In this regard, the quest for an effective adjuvant treatment with a robust advantage on the outcome of resected rectal cancer remain an important task for gastrointestinal oncologists.

In Japan, mesorectal excision is standard surgical procedure. Radiotherapy is not routinely performed as adjuvant therapy.

In Japan, adjuvant therapy after resection of colorectal cancer was developed primarily using oral fluorinated pyrimidines (O-FPs). A meta-analysis of three old trials (Sakamoto *et al*, 1999) and a more sophisticated analysis of four recent pivotal randomised trials (Sakamoto *et al*, 2004) demonstrated a statistically significant benefit of O-FPs on the outcome of colorectal cancers over surgery alone. However, the survival benefit shown in that meta-analysis was more pronounced in colon cancers. The risk reduction in terms of rectal cancer was only 8% and the result of those previous meta-analyses that analysed various types of oral fluorinated pyrimidine clinical trials was not sufficient to show a significant effect on survival.

Uracil–tegafur (UFT) is one of the O-FPs. In colon cancer, the majority of recurrences occurred in the liver, whereas in rectal cancer many recurrences occurred in the lung and locally in addition to the liver. Treatment effect may thus differ between colon cancer and rectal cancer. As the previous meta-analysis, two trials of UFT in patients with rectal cancer have been reported. The present study focused on rectal cancer, which lacked a clear-cut survival benefit in our previous meta-analysis. Unlike oral fluoropyrimidines such as carmofur and capecitabine, the formulation of UFT includes a dihydropyrimidine dehydrogenase inhibitor (Diasio, 1999), designed to enhance the bioavailability of FU. This combination of uracil and tegafur was shown, in an animal tumour system, to increase the anti-tumour activity compared with tegafur alone (Ooi *et al*, 2001). UFT also produced an enhanced intratumoural concentration of fluorinated pyrimidine, 5–10 times greater than that achieved with Tegafur alone (Fukunaga *et al*, 1987). Preclinical studies established that the optimal molar ratio of uracil to Tegafur is 4 : 1, which resulted in the highest 5-FU tumour: blood and tumour: normal tissue partition coefficients (Kawaguchi *et al*, 1980). UFT has now been clinically tested for lung cancer (Kato *et al*, 2004), breast cancer (Noguchi *et al*, 2005), and for gastric cancer (Kinoshita *et al*, 2005) in an adjuvant setting in Japan. Recently, UFT has also been tested in Western countries, regarding its efficacy for both advanced and curatively resected colon cancer (Carmichael *et al*, 2002; Douillard *et al*, 2002; Lembersky *et al*, 2006).

Here, we present an individual patient data meta-analysis of five centrally randomised trials recently performed in Japan to compare rectal cancer patients treated with UFT, with the surgery-alone control group. This meta-analysis includes data from more than 2000 patients and therefore provides a more reliable assessment of the effect of UFT on the survival, disease-free survival (DFS), and local relapse-free survival (LRFS) of the patients with rectal cancer than is available from any of the individual studies.

## PATIENTS AND METHODS

### Selection of trials

Trials that randomly assigned patients to either long-term (12 months) administration of UFT or surgery-alone treatment after curative resection of rectal cancer were eligible for meta-analysis. The randomisation technique used in these trials was the centralised randomisation that precluded the possibility of prior knowledge of the treatment to be allocated.

Five relevant trials identified as Japanese Foundation for Multidisciplinary Treatment of Cancer (JFMC) 7-1 (Kodaira *et al*, 1998), JFMC15-1, JFMC15-2 (Watanabe *et al*, 2004), Tokai Adjuvant Chemotherapy Study Group for Colorectal Cancer (TAC–CR) (Kato *et al*, 2002), and National Surgical Adjuvant Study of Colorectal Cancer (NSAS-CC) (Akasu *et al*, 2006) were included in the meta-analysis involving a total of 2091 patients. In trials JFMC7-1, JFMC15-1, and JFMC 15-2, patients who were randomly assigned to the experimental group received intravenous mitomycin C (6 mg m^−2^) at 1 week and once monthly for 6 months. In the JFMC15-1 and 15-2 trials, patients who were randomly assigned to the experimental group additionally received an induction course of intravenous 5-FU (250 mg daily^−1^) during 7 postoperative days (Table 1).

### Protocol and data collection for the meta-analysis

In December 2003, a protocol for the meta-analysis, describing the study rationale, statistical methods, and guidelines for publication, was distributed to the principal investigators of the five trials. Investigators were asked to provide individual data for every randomised patient, whether eligible or not, assessable or not, and properly followed up or not. Items requested for every patient were as follows: patient identification, date of surgery, eligibility, allocated treatment by random assignment, age, sex, primary tumour site, Dukes' stage, induction chemotherapy, dates of recurrence, death, or last visit. Disease-free survival was calculated from the date of surgery to the date of recurrence, second primary cancer or death, whichever occurred first. Survival was calculated from the date of surgery to the date of death, regardless of the cause of death. Local relapse-free survival was calculated from the date of surgery to the date of local recurrence. Data from patients with only distant recurrence and those who were died without recurrence were censored. Patients enrolled in these trials had been followed up for 5–7 years. Toxicity data were not collected, because detailed analysis of side effects can be found in the published reports of the individual trials (Kodaira *et al*, 1998; Kato *et al*, 2002; Watanabe *et al*, 2004; Akasu *et al*, 2006).

All investigators and the Clinical Trial Committee of all the trials agreed to join in the meta-analysis. Individual patient data were received by the independent secretariat by February 2004 and October 2006.

### Pretreatment patient characteristics

All 2091 patients had curatively resected rectal cancer without evidence of distant metastasis by diagnostic imaging criteria or by macroscopic examination of the abdominal organs during surgery. Patients with severe postoperative complications were excluded from all trials, as were patients with any previous chemotherapy or radiotherapy or with a synchronous or metachronous second cancer. Median patient age was 61 years at the time of random assignment. The male/female ratio was approximately 3 : 2. Performance status was less than 2 on the Japan Clinical Oncology Group scale for all patients.

### Statistical analysis

The method used for the meta-analysis and the format for the presentation of the results have been described in detail elsewhere (Advanced Colorectal Cancer Meta-Analysis Project, 1992). All analyses were based on individual patient data. Treatment effects on DFS, LRFS, and survival were first estimated within each trial and then combined using classical meta-analytic methods (Colorectal Cancer Collaborative Group, 2001). Treatment effects were displayed as hazard ratios. These ratios were estimated by univariate Cox's proportional model as relative risks of having an event in the UFT group as compared with having the same event in the surgery-alone control group. A ratio less than unity indicates benefit from UFT, and this benefit is statistically significant when the 95% confidence interval (CI) of the ratio does not include unity. The overall effect of treatment was assessed through a *χ*_1_^2^ d.f. and the heterogeneity between five trials through a *χ*_4_^2^ d.f. (Colorectal Cancer Collaborative Group, 2001). Additional analyses were carried out to determine which of the following prognostic features, if any, were predictive of the treatment effect: Dukes' stage (A *vs* B *vs* C), sex (male *vs* female), and age (three groups of increasing age). Tests for interaction were applied to detect departures from the homogeneity of treatment effects. Multivariate analyses were performed with the use of the Cox proportional hazards regression model for DFS, LRFS, and survival to assess the robustness of the observed effects to adjustments for important covariates and the magnitude of interaction between treatment effect and covariate (Advanced Colorectal Cancer Meta-Analysis Project, 1992). All *P*-values resulted from use of two-sided statistical tests. The significance level was set at 5% for all tests.

## RESULTS

### Survival

**Figure 1:**
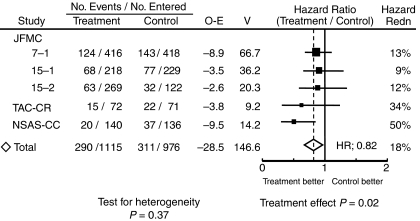
Survival hazard ratios by individual trial (Abbreviations: O/N=observed number of events/number of patients; O–E=Observed minus Expected number of events; V=variance of (O–E); Hazard Redn=hazard reduction; SE=standard error of hazard reduction).

Survival hazard ratios for all the trials are presented in Figure 1. The overall hazard ratio was 0.82 (95% CI, 0.70–0.97; *P*=0.02) with no significant heterogeneity between the treatment effects in different trials (*χ*_4_^2^ for heterogeneity=4.31; *P*=0.37). UFT showed significant effect on survival of curatively resected rectal cancers with a 5-year survival benefit of approximately 5%.

**Figure 2:**
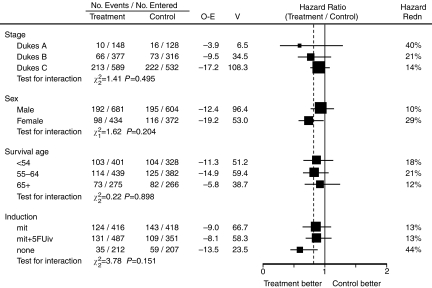
Survival hazard ratios by patient and treatment characteristics (Abbreviations as in Figure 1).

Figure 2 shows the breakdown of the survival hazard ratio stratified by various patient characteristics. There was a slight trend toward larger treatment benefits in earlier Dukes' stages (Hazard ratio; Dukes' *A*=0.60, Dukes' *B*=0.79, Dukes' *C*=0.86) but heterogeneity tests did not show any significant difference (*χ*_2_^2^=1.41; *P*=0.495). There was no statistically significant difference in sex (*χ*_1_^2^ for interaction=1.62; *P*=0.204) or age (*χ*_2_^2^ for interaction=0.22; *P*=0.898).

**Figure 3:**
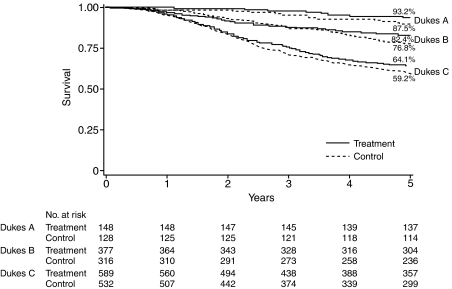
Survival curves by tumour stage and by treatment.

Figure 3 shows survival curves by treatment and disease stage. These curves confirm the hazard ratio analysis shown in Figure 2 and point to favourable effects of UFT in all Dukes' stages.

### Disease-free survival

**Figure 4:**
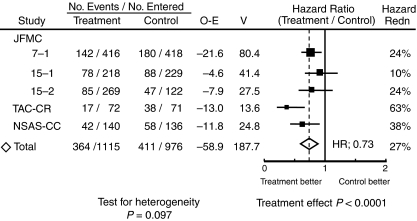
Disease-free survival hazard ratios by individual trial (Abbreviations as in Figure 1).

Disease-free survival hazard ratios are presented in Figure 4 for all the trials. These figure show a somewhat larger effect of treatment on DFS than on survival, with an overall DFS ratio of 0.73 (95%CI, 0.63–0.84; *P*<0.0001) with a 5-year DFS benefit of 9.7%, but demonstrating some heterogeneity among the treatment effects in different trials (*χ*_4_^2^ for heterogeneity=7.85; *P*=0.097). Additionally, random effect model assuming the variation between trials was applied. The results of the random effect model still revealed highly significant differences owing to the relatively high effect in TAC–CR and NSAS-CC trials.

**Figure 5:**
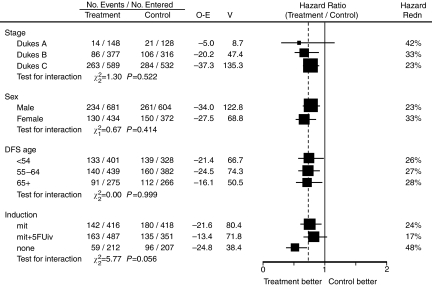
Disease-free survival hazard ratios by patient and treatment characteristics (Abbreviations as in Figure 1).

Figure 5 lists the DFS hazard ratios by various patient and treatment characteristics.

**Figure 6:**
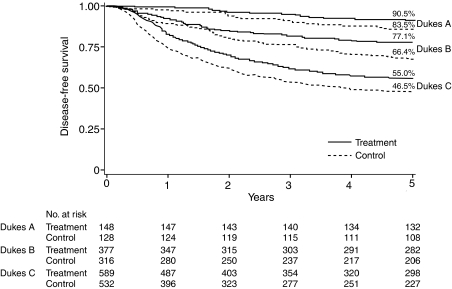
Disease-free survival curves by tumour stage and by treatment.

Figure 6 shows DFS curves by treatment and disease stage. These curves again point to benefits of UFT in Dukes' A, B and C stages. Roughly identical effect extended across all Dukes' stages: the DFS benefits at 5 years in terms of risk reduction were 0.42, 0.33, 0.23.

### Local relapse free survival

The overall hazard ratio was 0.68 (95%CI, 0.53–0.87; *P*=0.0026), and demonstrating some heterogeneity among the treatment effects in different trials (*χ*_4_^2^ for heterogeneity=8.82; *P*=0.0658). UFT also showed significant effect on LRFS of curatively resected rectal cancers.

## DISCUSSION

Extensive preclinical and clinical research led to the optimisation of 5-FU administration, with 5-FU bolus in combination with LV as standard therapy both in metastatic disease (Advanced Colorectal Cancer Meta-Analysis Project, 1992) and after curative resection of Stage III (Dukes' C) colon cancer (International Multicentre Pooled Analysis of Colon Cancer Trials (IMPACT) investigators, 1995; O'Connell *et al*, 1997; Wolmark *et al*, 1999). However, the toxicity of bolus 5-FU/LV regimen, especially the risk of haematologic toxicity and mucositis, could not have been negligible.

Continuous-infusion 5-FU modulated by LV, utilised mostly in European countries, showed somewhat better efficacy and definitely better tolerance than bolus 5-FU in advanced diseases (de Gramont *et al*, 1997; Meta-Analysis Group In Cancer, 1998a, 1998b; Schmoll *et al*, 2000). In the adjuvant setting, one of the continuous regimens (LV5-FU2) was shown to have low toxicity than the bolus regimen, but no difference was shown in terms of survival (André *et al*, 2003). Recently, combination of continuous 5-FU/LV and oxaliplatin (FOLFOX 4) was demonstrated to have significant effect on DFS, and is now considered as the standard adjuvant regimen for colon cancer in the Western world.

The recent development of O-FPs has therefore opened new perspectives. Oral fluorinated pyrimidines may mimic continuous regimens without its technical inconvenience and deterring patients' quality of life. In patients with advanced colorectal cancer, the efficacy of UFT (typical and most prescribed O-FP) plus oral LV (Carmichael *et al*, 2002; Douillard *et al*, 2002) or of capecitabine alone (Hoff *et al*, 2001; Van Cutsem *et al*, 2001) seems comparable in terms of the efficacy with significantly less significant severe haematologic toxicities and/or stomatitis. The risk of severe hand-foot syndrome is lower in UFT than with capecitabine, but the risk of severe diarrhoea and other gastrointestinal symptoms is higher in UFT and in UFT/oral LV treatment for Western patients.

In Japan, UFT have been administered for many years especially for patients with curatively resected colorectal cancers. For some unknown reason, severe gastrointestinal toxicities are much less frequent in Japanese patients, and patients usually prefer oral chemotherapy especially in an adjuvant setting (Borner *et al*, 2002).

Furthermore, with regard to rectal cancer, it is a difficult objective for a clinical trial to accrue enough patients, compared to colon cancer, and despite the fact that several attempts of determining a standard adjuvant treatment for rectal cancer, almost no clinical trial has succeeded in showing a relevant survival benefit of adjuvant treatment, except one with preoperative radiotherapy (Swedish Rectal Cancer Trial, 1997).

In this context, several Japanese groups conducted randomised clinical trials comparing UFT with surgery alone for curatively resected rectal cancers. Five such trials were identified after a meticulous search, and are included in the present meta-analysis. This meta-analysis was restricted to trials that had been randomised centrally and from which no patient had been excluded for any reason. It represents the largest series of properly randomly assigned patients receiving the single oral adjuvant O-FP agent, that is, UFT, for rectal cancer comparing with patients receiving no therapy after curative tumour resection.

This meta-analysis found a statistically significant benefit of UFT with regard to overall survival (OS) (hazard ratio=0.82; *P*=0.02) as well as DFS (hazard ratio=0.73; *P*<0.0001), and LRFS (hazard ratio=0.68; *P*=0.0026). As can seen by comparing the data in Figures 1 and 4, the data from the NSAS-CC and TAC–CR study show benefits that are, apparently, larger than the others. As shown in Table 1, the dosage and duration of treatment with UFT in the NSAS-CC and TAC–CR trials differed from those in the other three trials; the dose intensity of UFT was higher in the former two trials. Several studies have reported that a high-dose intensity of UFT improves survival in patients given postoperative adjuvant chemotherapy for gastric cancer (Sugimachi *et al*, 1997; Danno *et al*, 2001). The higher dose intensity of UFT in the NSAS-CC and TAC–CR trials may have influenced the outcomes.

Most of the Japanese rectal cancer patients did not receive pre- or postoperative radiotherapy in any of the trials. Although radiotherapy has been considered one of the standard adjuvant treatments in the Western countries, significant survival benefit has not been shown with reproducibility (Wolmark *et al*, 2000; Colorectal Cancer Collaborative Group, 2001). The ostensible advantage of adjuvant radiotherapy is to decrease local recurrence of rectal cancers. As compared with postoperative chemoradiotherapy, preoperative chemoradiotherapy does not improve OS, but inhibits local recurrence and reduces toxicity (Sauer *et al*, 2004). In our study, however, LRFS was also significantly better in the UFT group compared to surgery alone group. As far as our results are concerned, UFT might also be useful in preventing local recurrence in Japanese patients who usually do not receive radiotherapy in an adjuvant setting.

Also, there is still a debate whether adjuvant chemotherapy for early stage rectal cancer is feasible (Buyse and Piedbois, 2001). In terms of numbers needed to treat, these benefits imply that approximately 20 patients need to be treated for one more patient to survive 5 years, and approximately 10 to be treated for one fewer patient to suffer a cancer recurrence within 5 years, regardless of disease stage. Our results show that the therapy is beneficial in Stage II patients not only Stage III patients with nodal involvement (Mamounas *et al*, 1999; Gray *et al*, 2004). As for early stage disease, further investigations are needed to assess potential benefits of treatment because events were infrequent and hazard ratios were small.

Regardless of the disease stage and patient background characteristics, there is a need for further trials involving UFT and new agents that are effective in advanced disease, such as irinotecan, oxaliplatin, and monoclonal antibodies.

## Figures and Tables

**Table 1:** Details of the randomized controlled trials included in the individual patient data meta-analysis

**Category**	**JFMC7-1**	**JFMC15-1**	**JFMC15-2**	**TAC-CR**	**NSAS-CC**	**Total**
Additional chemotherapy	Mitomycin C	Mitomycin C+FU IV	Mitomycin C+FU IV	None	None	—
Radiotherapy	None	None	None	None	None	—
UFT dose/day	400 mg	400 mg	400 mg	400 mg	600 mg^a^	—
Period	12 months	12 months	12 months	24 months	12 months	—
Dates of accrual	1986–1988	1989	1990	1991–1994	1996–2001	—
No. of patients	834	447	391	143	276	—
Duration of accrual, months	35	24	24	36	54	—
						
*Sex, No. of patients (male–female ratio)*						
Male	521 (62.4%)	260 (58.1%)	244 (62.4%)	93 (65.0%)	167 (60.5%)	1285 (61.4%)
Female	313 (37.6%)	187 (41.9%)	147 (37.6%)	50 (35.0%)	109 (39.5%)	806 (38.9%)
						
*Duke's stage, No. of patients*						
A	135	67	62	12	0	276
B	326	175	139	53	0	693
C	373	205	189	78	276	1121
						
Median age	57	60	59	62	58	58
Upper age limit, years	70	75	75	75	75	—

JFMC=Japanese Foundation for Multidisciplinary Treatment of Cancer; NSAS-CC=National Surgical Adjuvant Study of Colorectal Cancer; TAC–CR=Tokai Adjuvant Chemotherapy for Colorectal Cancer; UFT=Uracil–Tegafur.

a 400 mg m^−2^ day^−1^ for 5 days every 7 days.
